# SHOW ME the evidence: features of an approach to reliably deliver research evidence to those who need it

**DOI:** 10.1186/s13750-024-00351-4

**Published:** 2024-11-21

**Authors:** John N. Lavis, Jeremy M. Grimshaw, Ruth Stewart, Julian Elliott, Will Moy, Joerg J. Meerpohl

**Affiliations:** 1 Department of Health Research Methods, Evidence and Impact, McMaster Health Forum, McMaster University, Hamilton, ON Canada; 2 Ottawa Hospital Research Institute and University of Ottawa, Ottawa, ON Canada; 3 Future Evidence Foundation and University College London and University of Cape Town, Johannesburg, South Africa; 4 Future Evidence Foundation and Monash University, Melbourne, VIC Australia; 5 Campbell Collaboration, London, UK; 6 Institute for Evidence in Medicine, Cochrane Germany, University of Freiburg, Freiburg, Germany

ED000170.

The world is poised for a step-change improvement in how we use evidence to address societal challenges.

Given the speed at which plans are being made to support this once-in-a-generation transformation, the Implementation Council of the Global Commission on Evidence to Address Societal Challenges developed a working version of the features of an approach to reliably getting research evidence to those who need it and achieved consensus among leaders from the Implementation Council, as well as the Alliance for Living Evidence (Alive) Council and Evidence Synthesis International (ESI).

Drawing an acronym from the first letter of each of the six features, the ‘SHOW ME the evidence’ features are as follows.

1. Support systems locally that use many forms of research evidence to help address local priorities
2. Harmonized efforts globally that make it easier to learn from others around the world
3. Open-science approaches that make it the norm to build on what others have done
4. Waste-reduction efforts that make the most of investments in evidence support and in research
5. Measured communications that clarify what we know from existing evidence and with what caveats
6. Equity and efficiency in all aspects of this work.

The 100 + contributing authors from across the ‘evidence synthesis and support’ world want to ensure that our future plans are firmly rooted in an agreed-upon summary of all we have learned together over these past four or so years, and to signal a mutual accountability among many of the key players involved in providing evidence support that we will each do our part in delivering on the promise that motivates these plans.

Given that much of the momentum for transformation is currently focused on living evidence syntheses and the infrastructure needed to support them, we give this form of evidence disproportionate focus here.

An even more diverse set of partners should be engaged in designing and executing an inclusive process for the refinement or even reshaping of these features over time, as well as their ongoing operationalization. This includes more types of decision makers, those working with more forms of evidence, and funders, as well as even more contributors from across the Global South.

1. **Support systems locally** that use many forms of research evidence to help address local priorities.

Every jurisdiction needs a reliable evidence-support system to get whatever forms of evidence are needed to address a local priority into the hands of those who need it, when they need it, in whatever form they need it, and with any required caveats about its currency, quality, and local applicability [1].

Locally can mean nations as well as subnational jurisdictions like provinces and cities. It can mean formal regional groupings of countries like the European Union and informal regional groupings of small countries with shared challenges. It can also mean systems, like the health or social-care system.

The forms of evidence can include research evidence from the ‘local’ context (e.g. data analytics, evaluation, and behavioural or implementation research), research evidence from around the world (i.e. evidence synthesis), and other types of information (e.g. horizon scanning and people’s lived experiences) and ways of knowing (e.g. Indigenous knowledge).

Addressing a local priority is ideally informed by an understanding of a problem (and its causes and alternative ways of framing it), options to address the problem (including those already in use at a small scale), implementation considerations, and how to monitor implementation and evaluate impact. Research evidence can inform such understandings alongside political and social insights.

Those who need research evidence can include government policy makers (from central agencies like Treasury, line departments like Education, and legislatures), organizational leaders (from both nongovernmental organizations and private companies), professionals (like nurses, teachers and veterinarians), and citizens (in the broadest sense of that term, and inclusive of undocumented individuals, as described in Sect. 3.6 of the Global Evidence Commission report 2022). They also need enablers, culture and capacity for evidence use.

Many decision makers need actionable insights from research evidence quickly when a ‘window of opportunity’ opens. Sometimes these windows are open for days, other times weeks, and rarely for longer. Evidence support can now work at the same speed as decision-making processes.

Some decision makers may want the evidence presented to them as ‘best buys’ (e.g. Global Education Evidence Advisory Panel), others by broad approach (e.g. Education Endowment Foundation), and still others by branded programme (e.g. IES What Works Clearinghouse).

Applicability can mean both for local contexts and for groups in a range of contexts, including groups most affected by historical and acute inequities.

2. **Harmonized efforts globally** that make it easier to learn from others around the world.

One aspect of evidence support that can now best be undertaken through harmonized efforts globally is to provide regularly updated summaries of what we have learned from around the world and how these findings vary by groups and contexts.

‘Living evidence synthesis’ is a relatively new approach to producing and maintaining these summaries [2]. The take-up of this approach accelerated during the COVID-19 pandemic and continues to accelerate. Artificial intelligence (AI) has enabled some of this acceleration, and can continue to do so if done safely and responsibly. We revisit AI in feature 6.

Groups of decision makers are beginning to come together to identify shared priorities and to call for living evidence syntheses that address these priorities. We are seeing this happen among United Nation (UN) agencies and their member states (through the Global SDG Synthesis Coalition), central agencies of government (through the Four-country commission), and international-assistance providers (indirectly through their chief economists or directly through their chief scientists). We foresee this happening in other areas like climate solutions and health technologies and in regions across the Global South. We hope the days will soon be gone when each organization separately commissioned or undertook its own rapidly outdated, often low-quality summaries, as well as when global-harmonization efforts are driven by a few dominant institutions or by a few high-income countries. We revisit this theme in feature 4.

Groups of living evidence synthesis producers are now working collaboratively to meet the needs of decision makers. Longstanding leaders in the evidence-synthesis field, like the Campbell Collaboration and Cochrane, have reorganized themselves to do so; The Alliance for Living Evidence (Alive) is testing a new collaborative model. Evidence Synthesis International or another ‘umbrella’ body could help to further accelerate these service-oriented collaborations [3]. Many groups are well positioned to share capacity in ways that ensure we achieve a distributed capacity for living evidence synthesis across low-, middle- and high-income countries.

Early movers and thought leaders are emerging among funders. For example, the Wellcome Trust has announced its intention to fund an evidence-synthesis infrastructure collaborative to support: (1) demand-side engagement through existing intermediaries; (2) data sharing and reusing; (3) safe and responsible use of AI; (4) methods and process innovation (e.g. related to equity considerations, context specificities, and feedback loops to primary researchers); and (5) capacity sharing through existing platforms. Such organizations are well poised to bring together a broad coalition of funders to invest in evolving suites of living evidence syntheses in areas prioritized by decision makers, and to invest in ways to serve actionable insights for diverse decision makers, sectors, regions, and languages. They are also well poised to make the case for sustained funding of national evidence-support systems.

We have witnessed some other aspects of evidence support be undertaken through harmonized efforts globally. Step-change improvements in data analytics across broad areas of human development, in modelling of climate change, in evaluations of multilateral institutions, and in health guidance, among other advances, did not come about by chance. Whether implicit or explicit, the five elements of a collective-impact approach have been used to sustain what’s going well – including in the transition to ‘living’ versions of many of these forms of evidence – and to prioritize and implement efforts to improve: (1) a common agenda (e.g. sustainable development goals or shared domestic priorities); (2) shared measurement systems and public reporting; (3) mutually reinforcing activities; (4) continuous communications; and (5) a strong and independent backbone function that supports the other four elements [4].

We urgently need to apply a collective-impact approach to living evidence syntheses. Contributors to the enterprise can be judged by whether their actions align with this approach. We also need to agree on flexible criteria for starting living evidence syntheses and for modifying and discontinuing them as context, issues and evidence evolve.

In time we also need to apply it to forms of evidence that haven’t yet benefited from global coordination and, most critically, to improving intersections among the many needed forms of evidence. The latter will require new forums with a demand-side orientation and a commitment to learning and working across forms of evidence, sectors and geographies, as well as new governance mechanisms.

3. **Open-science approaches** that make it the norm to build on what others have done.

A powerful enabler of evidence support is open data, particularly data that can be extracted from existing evidence and that can help with understanding its currency, quality, and local applicability.

Such data can be extracted once or – in the case of risk of bias and other quality assessments – be created once, and used many times. Consider the case of an evidence-support unit in a given country that is asked to summarize what has been learned from around the world about climate solutions that would be relevant to that country. That unit could be able to turn to a living evidence synthesis, access the data from studies conducted in its own country and relevant comparator countries and from studies examining interventions relevant to its own country, critique and correct the data where appropriate, and prepare a highly contextualized summary about what we know and don’t know, and with what caveats.

While this is already being done without delay at a small scale because of the generosity of a small number of living evidence synthesis producers, it can be the ‘new normal’ for all such producers. Making it so will mean finding new, sustainable funding for those groups whose data help them generate the revenue they need to do what they do, incentivizing all groups to contribute and acknowledging the contributions of those who do, unlocking the data in government-commissioned research that is not publicly shared or in UN evaluations and PhD theses that are not easily findable online, and assuring the quality of the data being shared.

More generally, all evidence producers can commit to the FAIR data principles of findable, accessible, interoperable and re-usable. They can also commit to the CARE principles for Indigenous data governance – collective benefit, authority to control, responsibility and ethics – or an appropriate alternative endorsed by their partners. Data-governance principles – data stewardship, data quality, data security, data privacy, and data management – are also important.

In time we also need to operationalize and sustainably fund other open-science approaches in how we provide evidence support to decision makers, including using open-source software, publishing in open-access publications (including the evidence maps and summaries that they often highly value), and sharing open-educational resources [5].

4. **Waste-reduction efforts** that make the most of investments in evidence support and in research.

Many labour-intensive aspects of providing evidence support are needlessly duplicated within countries (by different groups), across countries, and over time. An effort to address a local priority can begin with a profile of existing evidence from the ‘local’ context (e.g. data analytics, evaluation, and behavioural or implementation research) and existing synthesis of evidence from around the world, along with any caveats. Sometimes such a rapid evidence profile will give decision makers all that they need; other times it will identify existing work that can be built upon (e.g. an evidence synthesis that can be turned into a living evidence synthesis). Sometimes it will inform the creation of flows of new evidence (e.g. a rapid evaluation).

Much applied primary research does not address current or likely future decision maker priorities or does not have the design or methodological characteristics needed to add value in responses to likely questions about an area of priority. An effort to fund or undertake applied primary research can be justified based on a high-quality evidence synthesis of existing studies addressing the same question – ideally one that highlights how findings vary by groups and contexts – and follow available standards for the conduct and reporting of studies of that type. Answering implementation questions through existing administrative data is one of many other ways to reduce research waste. Replication studies – studies conducted using the same or similar methods as the original study to evaluate whether consistent results can be obtained – should continue to be encouraged.

Much applied secondary research (i.e. evidence synthesis) also does not address decision maker priorities or does not have the design or methodological characteristics, or the group and context sensitivities needed, to add value. An effort to fund or undertake an evidence synthesis can be justified based on evidence maps and protocol registries and following available standards. As we noted in feature 2, with an evolving suite of living evidence syntheses on the big questions of our time, we hope the days are gone when each organization separately commissioned or undertook its own rapidly outdated, often low-quality summaries.

5. **Measured communications** that clarify what we know from existing evidence and with what caveats.

Sharing what has been learned about a local priority means identifying the many forms of evidence needed to answer questions about the priority, looking in the right places for each form of evidence, summarizing what we have learned from each form of research evidence and where there are gaps and uncertainties in what we know, and providing any required caveats about the currency, quality, and local applicability of the available evidence. Messages need to be adjusted as the evidence, and the context and issues it is meant to inform, evolve over time.

Those engaged in communications and science advice need to recognize that their value accrues in significant part from their ability to respond to the priorities of decision makers with all of the available evidence (not just the evidence that they helped to produce) and to ‘show their work’ (i.e. provide the evidence on which they are basing their claims about what we know and with what caveats). Promoting one’s own work at the expense of all relevant work, and providing personal opinions without any transparency about their basis, are worth little.

Communicators and advisors also need to recognize that evidence is one of many inputs to decisions and to deliver their messages with corresponding humility. They need to recognize that evidence doesn’t speak for itself and that how we communicate can be as important as what we communicate. They need to support fact-checking and other efforts to counter misinformation using tactics that have been shown to be effective. They also need to contribute to (re)building trust in evidence-related institutions and more generally putting evidence at the centre of everyday life.

6. **Equity and efficiency** in all aspects of this work.

Providers and funders of evidence support can put equity, diversity and inclusion at the heart of all we do, including in governance, processes (including what data are captured about whom), and outcomes. This means sharing capacity, creating opportunities for co-creation, recognizing contributions, and using a ‘leave no one behind’ approach among diverse evidence producers, evidence intermediaries, evidence users (citizens, professionals, organizational leaders and government policy makers), and the ultimate beneficiaries of efforts to address societal challenges (citizens, as well as animals and our planetary boundaries). It also means including, sharing power with, and supporting leadership and organizations from the Global South, and more generally from groups most affected by inequities.

Providers of evidence support should also incorporate appropriate technology, including AI, in workflows, as performance metrics show it can be done efficiently and equitably, including without amplifying existing biases. As noted in feature 2, AI enabled some of the acceleration we have seen in the take-up of a living evidence approach. The safe and responsible use of AI will be key to further acceleration in this and other types of evidence support, and can be supported by ongoing research and guidance. Minimizing the environmental footprint of AI is also important.

Actions speak louder than words. If we are to deliver on the promise of a step-change improvement in how we use evidence to address societal challenges, then each of us needs to do our part to put in place the features of an approach to reliably getting research evidence to those who need it. Funding can enable it. Coordination can facilitate it. Reporting can celebrate it (and shame a go-it-alone ethos). Evaluation of our approaches can support continuous improvement. But only our actions can make it happen.

You may already be doing great work. Please keep it up.

If you want to embrace a new approach and don’t know where you can best fit in, check out the Global Evidence Commission’s work in formalizing and strengthening national (and subnational) evidence support systems, enhancing and leveraging the global evidence architecture, and putting evidence at the centre of everyday life. Or approach one of the Implementation Council members who you see doing exemplary work in your part of the world, in your type of role, in your sector, with your form of evidence, or with an innovation like AI-powered living evidence synthesis or storytelling that draws on both research evidence and Indigenous ways of knowing.

## Data Availability

There are no data used in this article.

## Declarations

### Provenance and peer review

This editorial was commissioned based on a proposal by the authors and was peer reviewed.
